# Supplementary material for: Soccer goalkeeper expertise identification based on eye movements
Source: PLoS One. 2021 May 19;16(5):e0251070. doi: 10.1371/journal.pone.0251070 (PMC8133470; doi:10.1371/journal.pone.0251070)
Supplement: Attachment — Submitted filename: Response to reviewer.docx [file pone.0251070.s002.docx]

Reviewer #2: Please see page 5.

We thank the reviewers for their valuable and constructive feedback, which have helped us to improve the manuscript. In the following we provide a point-wise response to the reviewer’s comments. Typos are corrected directly in the document (see “Revised Manuscript with Track Changes”). All changes are highlighted in the manuscript.


 General comments

1. One of my concerns with this study is that just because we can use this type of machine learning approach, it doesn’t mean we should. There is certainly a place for it, but I don’t think the authors did a good job of motivating its use.
2. It doesn’t tell us anything about what perceptual expertise actually is. Lots of variables are thrown in an algorithm but we don’t learn anything about what experts do differently. So what is the purpose?
3. The purpose can’t actually be identifying experts, because we would fare much better by just asking how often they play soccer.
4. While the authors discuss the benefits of VR for making perceptual-cognitive research better by enabling more realistic eye movements, their approach sort of misses the point of why this is useful.
5. The authors analyse eye movements in a way that they are completely disconnected from what the participants are viewing. Eye movements are meaningful in relation to the stimuli that the person is viewing (unless they are being used to infer a general state like anxiety, which is not the case here). Lets say, for instance, you find that experts are showing larger saccades. That finding is pretty meaningless when it is out of the context of what they were viewing. Unless the eye movements are coupled to the scene it is very hard to interpret what any of them mean or what they tell us about expertise. I just feel that this approach misses the benefit of doing this work in VR.

- *1) One major advantage of machine learning is its ability to work with big data. We added a paragraph to the introduction where we explain that we reached a point where we gain a lot of knowledge from high speed devices like eye trackers, but still use manual methods to infer high level knowledge from it. Machine learning is a consequent step since these techniques become stronger with more data. And therefore, allows experimenters to learn more about perception of athletes. Machine learning can also provide valuable insights on the importance of eye movement features to expertise. Since it is hard for human to describe a gaze behaviour by getting asked, machine learning is able to find latent feature and feature patterns, one can not simply ask. According to the reviewer’s feedback, we added a paragraph to motivate the usage of machine learning in our approach. We hope it is much clearer now.*
- *2) One disadvantage of machine learning is the explainability. Often, machine learning methods find patterns that are not that obvious for human to understand. But in this work we do not focus on the understanding of differences between expertise groups, since we first want to find out whether expertise can be classified with these methods. We use a lot of variables to have a higher chance of finding a subset that predicts expertise with a small amount of features. Not to find a subset that explains the differences, but allows a machine learning based classification of expertise. In a further work we want to focus on explainable differences.*
- *3) An expert goalkeeper does not need to have a high expertise in perceptual skills. Some only have good goalkeeper skills. Eye tracking reveals subconscious behaviour and therefore allows to investigate on non-obvious behaviour. Most players cannot properly tell where they are looking and why as subconscious processes are involved. With the combination of eye tracking and machine learning we can work on much deeper levels of perception. We can not ask athletes where did you look when, how long do you follow the ball, how important is the length of your smooth pursuit. This all happens in subconscious processes. For a training system we need to find the fine nuances in their gaze.*

*4) Stimuli in VR lead to an increase in external validity. This increases the probability that the participants will perform a natural gaze behaviour. Only in this way it is possible to test whether the level of expertise can be recognised on the basis of eye movements. We need realistic eye movements as at some point, since the motivation of our work is also to develop a diagnosis system, that can be used at home especially by young athletes to diagnose their expertise. These findings will provide a basis to build a training system in a next step to enhance perceptual expertise of young talents in a home training system.*

- *5) The experimental control of this study allows us to infer the expertise of these participants in a stimulus unrelated manner, but close to natural environment. You are right, eye movements are meaningful in relation to stimulus, yes. But as every participant saw the same videos we can abstract their behaviour. We pointed out, that a stimulus-oriented investigation might reveal more pronounced differences. But as every participant saw the same videos we can abstract their behaviour. Our samples are average values over one video trial. As we have a lot of features that describe the gaze behaviour, the differences can be reflected in more than one feature. We want to condense the amount of features to know how we can abstract the expertise based on average features of one video into expertise classes.*

*We do not think the findings are meaningless. Because we assume that participants act always more or less the same when the task is decision-making under high pressure. But you are right: Without the relation to the stimulus, it is really hard to interpret the features. As we stated, the smooth pursuits need to be investigated further, since we might have found a correlation between the movement of the players and the smooth pursuits. For the diagnostic system it is less important for us to know how the differences in gaze behaviour are, but more whether the differences can be used to differentiate participants into expertise classes.*

Specific comments:

6. L39 – I think this is a really key point - the assumption that gaze behaviour will be the same in VR. Which I am pretty confident has not been properly tested. I am in agreement that VR is a good way to examine perceptual-cognitive skills but the evidence that it elicits similar gaze behaviours is not there yet. Indeed, the extent to which a VR environment elicits realistic gaze behaviour is likely to be environment specific, and would be referred to as the ‘psychological fidelity’ of the environment.
Both these papers discuss this issue:
-Gray, R. (2019). Virtual environments and their role in developing perceptual-cognitive skills in sports. Anticipation and decision making in sport, 342-358.
-Harris, D. J., Bird, J. M., Smart, A. P., Wilson, M. R., & Vine, S. J. (2020). A framework for the testing and validation of simulated environments in experimentation and training. Frontiers in Psychology, 11, 605.

- *Changed and added psychological fidelity. Please see p.3, r. 42 ff*

7. L49 – I think ‘objective reproducible’ is debatable. Machine learning still depends on the decisions made by the researcher about how to treat the data. It not as assumption free as people would like to think.

- *Partly, this is true. But as long as the parameter set of the model is published too (as we did), it is easy to reproduce the model. The paper needs to describe the way parameters were set. Usually “why” cannot be answered, as this is mostly an empirical search or grid search.*

8. L57 – perhaps you could explain more how it would lead to training. This section is a bit vague. Adjusting training difficulty to the level of expertise of the trainee is not something new. That’s how training always works.

- *We moved the description of the training system to implications section and added a more detailed description on how this can lead to a training system.
  p. 26 r. 533 ff*

9. L90 – the novice group seems quite disparate. It includes both soccer players and non-soccer players. Why was this choice made? Either low level players or non-players might make sense, but a mixture is an odd design choice.

- *That’s a good point. Actually, the novices had once played either in a youth league or never at all. This means, the novice group contains participants who have no training on a regular basis and are not participating in competitions. We added a table containing attributes of the single groups. See p. 7, table 1*

10. L113 – I know the software calculates the fixations etc, but it would be useful to add what the parameters were. E.g. if you are interested in fixations what constitutes a fixation in this case, what constitutes a saccade etc. How was smooth pursuit calculated? I have used BeGaze software but do not recall it calculating smooth pursuit.

- *We added more details on this procedure in p. 7 and 8. The software first calculates the saccades based on the peak threshold, which means the minimum saccade duration (in ms) varies and is set dependent on the peak threshold default value of 40°/s. In a second step the software calculates the fixations. Samples are considered to belong to a fixation, when they are between a saccade or blink. With a minimum fixation duration of 50 ms we reject all fixations below this threshold. As there is no generally applicable method for detection of smooth pursuits, this kind of event is included and encoded as fixations with longer duration and longer dispersion in the fixations, too. We marked fixations with a fixation dispersion of more than 100 px as smooth pursuits. By doing this, we split fixations into normal length fixations and long fixations which we consider to be and call smooth pursuits. This threshold is an empirical value based on the sizes of the players as main stimuli in the video. The following section describes the steps that are necessary to train a model based on these eye movement features*

11. I also think there needs to be some discussion of what all these metrics mean. There must have been a rationale for including them and it would be useful to explain to the reader how to interpret the measures. For instance what does average saccade deceleration show about the perceptual behaviour of the performer? What does it mean in the context of this task? Otherwise they are just numbers.

- *We added a paragraph in the manuscript where we talk about the different metrics. Explaining all features would be too much for the paper. We took only the most frequent features and explained them in a more detailed way. As we start with a brute force method and find the important features during the training, we only consider the most frequent features. These features have the highest importance for the model to predict. Please see p. 22 for a discussion.*

12. L132 – please report how many trials (and %) were lost. And were any participants totally removed? (I do see that some of this is reported below, but an overall figure would be useful)

- *No participants were removed. We added a finer description and a table on p. 10. See table 2 for an overview.*

13. L151 – what do you mean by samples 7, 8, 14-16, 18-20? Are these participants? Or parts of every trial? Either way it seems concerning that these were lost.
There is lots of details on what an invalid saccade amplitude value is, but you haven’t even described how a saccade was identified as a saccade. Similarly there is little detail on how fixations were treated, filtered etc.

- *Please see Method chapter for updated description about saccades and fixations (p. 4, r. 82 ff . and p.8, r. 181ff).*
- *These samples are gaze signal samples (see p.12, Fig. 3 caption)*

14. L184 – how can this work when there were only 8 intermediates?

- *The number 8 is correct. Since we only have 10 intermediates we use 8/13 novices, 8/10 intermediates and 8/12 experts. There was a typo at another point saying there were only 8 intermediates. So we have 2 intermediate left for validation, p.13 r. 266 ff.*

15. I am not familiar with how to gauge power for machine learning models but I think some discussion of why this number of participants and trials was appropriate is needed. 8 in a group would generally be considered quite low.

- *The chance level for 3 classes is 33.33 %. A system that would only guess the correct class would usually end up with an accuracy of about 33.33 %. All three models show a much better performance. Not in each run, but on average. But even the worst classification is over 20 % higher than chance level. Machine learning models for classification expertise are usually considered as good performing, when their average accuracy is between 70 % and 80 %. A statement about the performance of a model with lower than 70 % accuracy depends on the task and how much data is available. Sometimes there are only a few people in the whole world, considered as experts.*
- *We are aware that the sample size might restrict the conclusions, that can be drawn but the number of experts is strictly limited. We were very lucky to get 12 experts in our study. Please note that our method has no problems in detecting experts. The problem is more on classifying novices (mentioned in the discussion).*

16. L295 – how are these p values corrected for the multitude of comparisons that are made?

- *The desired alpha level is 0.05 (5%). We corrected this level with the Bonferroni method by dividing the desired alpha level by the number of tests (46). This results in a significance level of 0.0011 (0.11%).*

17. L298 – this is a real over-interpretation of the standard deviation values when the value for the intermediate group might just be higher because the sample is smaller (indeed that is how a standard deviation works).

- *We stated on p. 24, r. 444 ff. that it would be wrong to draw a conclusion about the standard deviation and added some other possible sources.*

18. Fig 7 – it would be useful to put some detail in the figure legend about what is plotted. Like what are those error bars?

- *Added a caption to figure 7.*

19. Also the prediction interval seems to be pretty wide; its somewhere between perfect and 50%. Is this a reflection of the limited size of the data set?

*– No it is no reflection of the dataset. On a binary decision, 50% is chance level. 50% would be the
 accuracy, if the model would guess. Everything better than this, is considered acceptable.
 Everything over 70% is considered good. We added this discussion to the manuscript as well.
 Please see p. 22, r. 404 ff.*

*20.* Your results focus on the comparison the different models, which all provide a fairly similar level of prediction, but I think it would be more useful to discuss what this means in real terms. Whether one model is better than another is really much less relevant that considering what a good level of prediction is in this context? What would be a good/acceptable/poor level of accuracy? What level of accuracy is needed for the type of training you discuss? With these types of statistical approaches it is very easy to get abstracted from the actual context (one of the contextual issues being that I can get 100% accuracy by just asking them).

- *The idea of the different models is to understand that we can build a model, which is as good as
the all feature model with much less features. As we can use less features, we get the information of which features of the gaze behaviour are important for a SVM to predict well.* *We added a
paragraph to explain this part to page p. 16, r. 310 ff. Another point to consider is the
following: a goalkeeper expert (who is classified as expert because of the league he is playing in),
does not need to have a very good gaze behaviour. Maybe he is playing in this high league because
he is really good in keeping the ball off the goal but has problems in deciding how to continue the
game after a return pass, because of a bad gaze behaviour. Our diagnosis system could discover
such athlete. The integration of the goalkeeper into the game as a player not just as a goalkeeper is
becoming more and more important these days, which makes it necessary to understand their gaze
behaviour and build a training system to overcome these problems. Still the main task remains
keeping the ball off the goal.*

21. L298 – this could just be a function of your definition of ‘intermediate’. What ages are the intermediates? (More detail needed in the participant section) because it sounds like they might be adults, in which case they might have more soccer experience than the experts. Similarly some of the novices could have had plenty of experience, but just be less good.

- *We added an overview of the participants. Please see p. 7, table 1.*

22. L307 – no they don’t have longer to process information. They use a visual strategy of longer fixations because it is more effective for extracting information because of saccadic suppression.

- *Right. Corrected. Bad translation. P. 24, r. 448 ff.*

23. It is surprising that well established features of perceptual-cognitive expertise, like longer fixation durations, were not found. Might be worth discussing.

- *Actually, it is difficult to say, since we splitted the fixations and smooth pursuits by a certain threshold. If one has a look at all the fixation values alone, there might be the same findings. But since we focused on this split into small fixations and smooth pursuits, we can not properly confirm the common findings. We just mentioned it only indirectly by the saccades behavior (p. 24, r. 450 ff. ).*

24. L328 – I don’t see how having a faster maximum saccade (which is not actually the same as faster saccades as you state) means that experts adapt themselves to the situation well. They almost certainly do, but it’s a huge leap to say that this data shows that.

*- You are right. This conclusion is too far away. We removed this conclusion, since we can not
 proof it properly.*

25. L331 – where has this data come from? Is this just an observation of the experimenter during testing?

*- Yes, as it is written on p. 24, r. 464 ff. We added some sentences about theoretical goals in
 football training, that fits to this observation.*

26. This kind of data that is linked to actual stimuli is much more meaningful for understanding expertise than, say, standard deviation of peak saccade deceleration.

*- We did that in a further study with the same stimuli and will compare the results between a stimulus oriented and stimulus-independent approach. We also added a paragraph to state that it might be better to go with stimulus-oriented p. 25, r. 501 ff.*

27. It seems odd to start a discussion with the limitations. It would be more conventional to restate your research question, your hypotheses and then say whether the results supported them or not.

*- Thank you. We rearranged the discussion and conclusion sections.*

28. L349 to 354 – I don’t see how the findings have shown this in any way. You have not shown that the
gaze behaviour was similar to natural gaze behaviour, you haven’t even measured natural gaze
behaviour, so how can you conclude this?

*- Correct. We did not measure natural gaze behaviour. We wanted to point out that we used
 VR-glasses and participants told us they feel really immersed into the scene.*

*Reviewer 2:*

Dear Reviewer,

Thank you for your valuable comments on our manuscript. We appreciate your help to improve our work. In the following we want to address your concerns. Typos are corrected in the manuscript directly. Please have a look at “Revised Manuscript with Track Changes”.

29. The study presents the results of primary scientific research. The study presents some results of primary research; however, the quality of this research appears to be somewhat limited in sample size.

- *We are aware that the sample size might restrict the conclusions, that can be drawn but experts are busy people, so DFB could give us only enough time to record 12 of them. We were very lucky to get 12 out of overall 56 experts in our study. Also, please note that our method has no problems in detecting experts. The problem is more on classifying novices (mentioned in the discussion).*

30. Also, due to a lacking literature research, their novelty regarding the sports science field is
rather unclear (the novelty is there, I just want them to show it more clearly, more on this in
my following comment). The literature review is rather limited. Regarding the VR aspect, the merit of
choosing specifically that technology above others is somewhat explained. However, is rather surprising
to see that the utilization of VR in other sports fields such as tennis is introduced, while the
soccer specific applications are not.

- *We added some of your mentioned researchers to the introduction to demarcate ourselves.*

31. The subjects are divided in three levels of expertise. However, there is a significant difference between someone without any soccer experience and someone that plays in a low-ranked league. Being a bit more specific in their subject’s selection criteria would be good. Also, in relation to the participants I could not find the information about their age and gender. One could somewhat infer the ages of the expert group but not for the others. Is better to show the descriptive statistics of the participants if available.

- *Added more details about subjects. Added table containing age, training/week and active years (see p. 6).*

32. L108-L109 The criteria used to define "the option that has the highest probability to lead to a goal…" is unclear. If defined by experts then please provide some details or if it was based on related research please cite.

- *This was a wrong statement. We added an explanation.*

33. Generally, this article needs substantial work in the organization of the paper and proofreading. As a reference some errors are outlined below, but as the paper stands, general improvement is needed.

*- We organized the paper differently now by using a more common structure and we have a native speaker proof reading it.*

34. Finally, the structure of the paper seems a bit unconventional. I suggest the authors carefully structure the paper in order to ensure logical progression of ideas. I feel the paper would flow well in a more traditional sense (introduction, methods, results, discussion). Integrating the Project Description section into Methods, then subdividing the VR and ML components of the system description as dedicated sub-sections could be an alternative.

- *Thank you for this feedback. We changed the structure of the paper and hope it is now much clearer.*
