# Supplementary material for: Soccer goalkeeper expertise identification based on eye movements
Source: PLoS One. 2021 May 19;16(5):e0251070. doi: 10.1371/journal.pone.0251070 (PMC8133470; doi:10.1371/journal.pone.0251070)
Supplement: Attachment — Submitted filename: Response to Reviewers.docx [file pone.0251070.s003.docx]

We thank the reviewers for their constructive feedback, which helped to further improve the quality of our manuscript. In the following, we provide a point-by-point response with our answers marked in blue.

Response to Reviewers:

“The use of references in the first few paragraphs of the introduction is quite infrequent, where one might expect background literature to be more regularly cited. Some more citations here about perceptual-cognitive expertise etc would be useful.”

We agree with the reviewer and added more related work to address perceptual cognitive expertise in the context of our work. (see line 3, 7, 8, and 78)

L13 – ‘a more highly developed perception,’ – should this be ‘a higher level of perceptual-cognitive skill’? Perception is really just the interpretations of sensory signals. I don’t think experts necessarily see anything differently, they just look in the right areas and interpret the information in a better way.

We corrected this statement in the manuscript accordingly (line 9).

L34 – please check, it sounds odd.

We changed the wording of the sentence (line 24).

L52 – CAVE is a form of virtual reality

We adjusted the wording to “4k 360° video in HMDs,…” (line 36-37).

L282 – I understand that definitions of smooth pursuit are difficult, but it would seem rigorous to justify your criteria with reference to previous work, rather than what would appear to be some arbitrarily chosen criteria.

Since we only have the dispersion in pixels, it is even harder to find a reference, as there are no information about the size of a pixel of the HMD in mm. We therefore stick to the size of 100 px which corresponds to the typical length of the routes between the players.

Table 2 – should column 3 be called ‘invalid trials’ instead?

This point is also corrected in the revised version (see table 2, line 229-230).

L440/441 – ‘the results of an intra-expert classification test to see whether inter-experts differences are smaller than than inter-class differences.’ I got confused here, although this may be my fault. Intra-expert classification test for inter-expert differences was confusing. Perhaps check this is the terminology you intended to use and also whether some of this terminology could be clearer. What is intra-expert? The within person variation for individuals in the expert group? (also typo with double than)L447 – Again, is intra-expert the right word here? It sounds like you are comparing experts and intermediates, which would be inter-something, would it not?L454 – intra instead of inter here, surely? Perhaps between-group/within-group terminology would be easier.

We changed the wording to gaze variation between experts/ groups and hope to have made this point clearer now (line 339-354).
